# Accurate LC Peak Boundary Detection for ^16^ *O/* ^18^ *O* Labeled LC-MS Data

**DOI:** 10.1371/journal.pone.0072951

**Published:** 2013-10-07

**Authors:** Jian Cui, Konstantinos Petritis, Tony Tegeler, Brianne Petritis, Xuepo Ma, Yufang Jin, Shou-Jiang (SJ) Gao, Jianqiu (Michelle) Zhang

**Affiliations:** 1 Department of Electrical Engineering, University of Texas at San Antonio, San Antonio, Texas, United States of America; 2 Center for Proteomics, Translational Genomics Research Institute, Phoenix, Arizona, United States of America; 3 Keck School of Medicine, University of Southern California, Los Angeles, California, United States of America; UGent/VIB, Belgium

## Abstract

In liquid chromatography-mass spectrometry (LC-MS), parts of LC peaks are often corrupted by their co-eluting peptides, which results in increased quantification variance. In this paper, we propose to apply accurate LC peak boundary detection to remove the corrupted part of LC peaks. Accurate LC peak boundary detection is achieved by checking the consistency of intensity patterns within peptide elution time ranges. In addition, we remove peptides with erroneous mass assignment through model fitness check, which compares observed intensity patterns to theoretically constructed ones. The proposed algorithm can significantly improve the accuracy and precision of peptide ratio measurements.

## Introduction


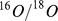
 labeling is often coupled with Liquid Chromatography-Mass Spectrometry (LC-MS) for protein quantification. In such experiments, two 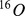 atoms are typically replaced by two 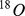 atoms by enzyme-catalyzed oxygen-exchange in the presence of 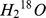 in the heavy sample [1]. This method has the following advantages [2]: 1) It does not target peptides containing particular amino acids and does not require an additional affinity-based step for labeled peptide enrichment; 2) It is amenable to clinically relevant samples; and 3) It is well suited for amount-limited samples. Due to these advantages, 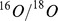 can be used in clinical or time critical applications where more accurate metabolite labeling methods [3] cannot be applied. However large experimental variation exists [4] in 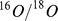 data since samples are combined after the digestion stage. This poses a great challenge in data processing, which is the focus of this paper.

Before detailed discussion, we need to clarify the definition of a few terminologies. An LC-MS peptide feature is the series of two dimensional (retention/elution time – mass/charge (m/z)) signals registered by a single charge variant of a peptide at different isotope positions. If we further integrate the 2D signals within narrow windows around the center m/z values of peptide isotopes, the feature is reduced to a group of LC peaks at different isotope positions. We further define peptide features of identical peptides in different replicates as corresponding ones.

In LC-MS, many co-eluting peptides have overlapping LC peaks, which will significantly increase the variance of measured Heavy/Light ratios (HLR)s between labeled and unlabeled peptides. Although numerous algorithms [5]–[7] have been proposed for separating overlapping peaks, they are generally computationally expensive and difficult to adopt. In this paper, we consider the relatively simpler problem of LC peak boundary detection (BD), which aims at removing LC peak segments that have been corrupted by co-eluting peptides. Besides BD, there is the issue of peptide mass ambiguity when the monoisotope mass is unknown. Wrongly assigned mass will lead to increased quantification error. Although these problems plague all LC-MS quantification methods, they severely affect the applicability of 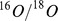 labeling, which has high variance due to sample preparation [4].

LC peak boundary detection determines which scans should be included in the LC peaks of a peptide feature. Current software packages do not employ accurate boundary detection especially on crowded Extracted Ion Chromatograms (XICs): QUIL [8] and ProteinQuant [9] determine LC peak boundary by the apex and the full-width-half-maximum (FWHM) of a peak; MsInspect [10] and SuperHirn [11] use thresholds; ASAPRatio [12] and MapQuant [13] use peak apex and FWHM; and MaxQuant [14] uses local minima after XIC smoothing. These algorithms cannot guarantee the exclusion of noise or interference-corrupted scans. Recently in MRCQuant [15], an algorithm that uses MS peak templates extracted at the highest isotope positions is proposed for boundary detection. However, MRCQuant is designed for low resolution label-free LC-MS applications, where there is significant noise and interference. The boundary detection method in MRCQuant is not effective for keeping the entire intensity pattern consistent within the boundary of LC peaks of 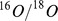 data, because it only uses MS peak templates at the highest isotope positions.

Given the importance of interference removal, we propose a simple but effective method for boundary detection. The proposed method is based on the observation that in most cases, a peptide has a consistent intensity pattern on scans within its non-corrupted LC peak segment. Interference from co-eluting peptides can be detected once such consistency is violated. The consistency of intensity patterns is calculated using the Kullback-Leibler (KL) distance [16]. Our testing results show that most peptides can be accurately quantified even if their LC peaks are partially corrupted by co-eluting peptides.

To address the issue of peptide mass ambiguity, we propose to use model fitness check (MFC) to remove peptide features with erroneously assigned masses. Given an assumed peptide sequence/mass and estimated heavy and light peptide intensities, we first construct a theoretically predicted intensity pattern. Subsequently, the predicted intensity pattern will be compared to the observation. If a match is found, the assumed peptide sequence/mass will be accepted. While the idea of comparing natural isotope patterns to observation data has been used for peptide identification in software like msInspect, the idea of using predicted intensity patterns constructed from estimated heavy/light intensities and isotope patterns for MFC has not been used previously. More background information on isotopic distribution can be found in [17].

In the proposed algorithm, the required input includes an LC-MS data file and a list of mass and charge (m/z) values, which can be compiled from currently or previously identified peptides through tandem MS with or without elution time information. The algorithm will perform BD and MFC for each peptide entry first, and after which, existing quantification methods [18]–[22] designed for 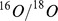 data can be applied.

We test the proposed algorithm based on data collected from two experiments using a Thermo LTQ Orbitrap Velos ETD mass spectrometer, and a Waters SYNAPT G2 Time-of-flight (TOF) mass spectrometer. While no tandem MS scans are collected on TOF, tandem MS scans are collected on Orbitrap, which provide a list of peptide m/z and elution time values after peptide identification.

In the first experiment, cells in the same biological condition are separated into two parts. Then they are labeled and combined at predefined ratios of 1∶1, 2.5∶1 and 5∶1 to create samples for checking the overall performance of the proposed algorithm. We evaluate the receiver operating characteristic (ROC) curves and show significant improvement of the proposed algorithm comparing to a popular software that can process high resolution 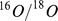 data, MaxQuant [14].

To further verify the proposed algorithm, cells in two biological conditions are labeled with 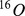 and 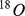 respectively in the second experiment, and equal amount of proteins from each condition are combined and analyzed. Technical replicates are collected on both instruments. This represents a typical scenario in biomedical research. Since peptide HLRs are not known in this case, we can not assess accuracy and precision by the mean and the standard deviation of measured HLRs. Instead, we employ two alternative measures, Normalized Mean Absolute Error (NMAE) for accuracy, and Log-Ratio-Difference (LRD) for precision. LRD is calculated by taking the difference between two 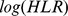 measurements of the same peptide. The variance of LRD can be attributed to the instrument and the data analysis process, but not the sample preparation process. Sample preparation causes a common deviation on the two measurements of the same peptide, which is canceled out when calculating the LRD. We have verified that NMAE and LRD are correlated with classical accuracy and classical measures, and they can be used on samples without predefined ratios for performance evaluation. Our experimental results show a significant improvement in NMAE and LRD by using the proposed processing steps on Orbitrap and TOF data.

We anticipate that the proposed algorithm can be incorporated into many kinds of LC-MS quantification software for significant improvement in quantification accuracy and precision.

## Data Collection

### Experiment one sample preparation

In experiment one, the samples from Human embryonic kidney 293T cells were divided into two groups. The two groups of samples were lysed in 8 M urea, and 50 mM ammonium bicarbonate (pH 8.3). The lysates were subjected to centrifugation at 13,000 rpm for 20 minutes and the supernatants were collected. The two samples were then denatured in 8 M urea, reduced using 10 mM dithiothreitol (DTT), alkylated with 30 mM iodoacetamide, and digested with trypsin (using an enzyme to protein ratio of 1∶50) at 37°C overnight. The samples were desalted with Sep-Pak cartridges, separated into two tubes and dried in a speedvac. The first sample was resuspended in 100 mL 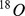-water (Purity >98%) containing 50 mM ammonium bicarbonate, 10 mM calcium chloride, and trypsin (1 to 50 w/w trypsin: peptide) pH 7.8. The second sample was treated in the same manner except that the 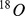-water was replaced with purified 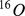-water. After incubation with shaking at 450 rpm for 5 hours at 37°C the labeling reaction was terminated by first boiling the sample for 10 minutes and then adding 5 mL of formic acid to further inhibit any residual trypsin activity. A bicinchoninic acid (BCA) assay was performed to determine peptide concentration. The two samples were combined equally or in selected ratios (1∶1, 2.5∶1, and 5∶1 Heavy/Light) and were subjected to reverse phase liquid chromatography (LC) followed by ETD-LTQ-Orbitrap Velos mass spectrometry (MS) analysis (see Experimental section for 1D LC and ETD-LTQ-Orbitrap Velos MS conditions in document S2).

### Experiment two sample preparation

In experiment two, the samples from Human embryonic kidney 293T cells were cultured in Dulbecco's modified Eagle's medium with 10% fetal bovine serum (FBS). One group was transfected with an expression vector expressing microRNA-K1 of Kaposi's sarcoma-associated herpesvirus (KSHV) while the control group was transfected with a vector for 48 h [23]. The sample preparation on these two groups of samples is the same as in experiment one. Equal amounts of 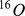 and 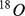 labeled samples were combined to obtain one sample. Two hundred micro grams of combined sample was fractionated into four fractions using strong cation exchange (SCX) (see Experimental section on strong cation exchange for LC conditions in document S2). The four samples were then subjected to reverse phase-reverse phase LC followed by ETD-LTQ-Orbitrap Velos MS and SYNAPT G2 MS analysis (see Experimental section for 2D LC and MS conditions in document S2).

### Tandem MS data processing and LC-MS quantification by MaxQuant

We download MaxQuant 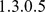 from the webpage www.maxquant.org, which uses Andromeda for tandem MS search. International Protein Index (IPI) human database version 3.83 is selected as the source of protein sequences. We set MS1 tolerance to 20 ppm for the first search and 6 ppm for the main search. We set MS/MS tolerance to 20 ppm, peptide FDR to 0.01, and select 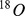 as the heavy label. We select Oxidation (M) and Acetyl (Protein N-term) as variable modifications, and Carbamidomethyl as the fixed modification. In database search, “minimum length of peptide” is set to 7, and “maximum missed cleavage sites” is set to 2. Peptide identification results are exported into text files and further imported into MatLab for quantification analysis. For further details of MaxQuant parameter settings, please visit the project website at http://compgenomics.utsa.edu/zgroup/boundarydetection/boundarydetection.html, where we provide screen shots of parameter settings.

## Data Model

Before further discussion, we would like to describe the data model used in this paper. Suppose a given peptide with mass 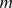 and 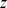 charges has sequence information, and based on which, we can theoretically predict its natural isotope pattern as 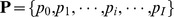 [24], where 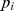 is the probability that the peptide has 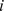 extra neutrons comparing to the monoisotope, and 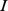 is the total number of isotopes considered. The corresponding m/z values of these isotopes are given by
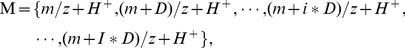
(1)where 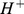 stands for the mass of the charge, and 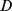 is the mass of an extra neutron.

Given an unlabeled peptide in 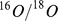 data, the signal of its heavy labeled version can be found at masses with 2 to 4 Daltons' shift with one or two incorporated 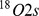. Suppose 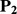 and 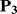 are isotope patterns of the peptide in heavy forms with one or two 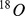 labels respectively, we can write
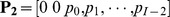


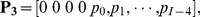
(2)in which, extra zeros are padded to reflect the mass shifts. In this paper, we consider a maximum of 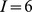 isotopes starting from the monoisotopic position of the unlabeled peptide. In this way, at least four and two isotopes of singly and doubly 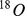 labeled peptides are included. Now we can express the observed peptide intensities in the 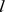
th scan as:
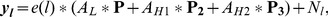
(3)where 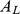, 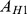, and 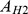 are the abundances of the unlabeled and labeled peptides with one or two 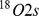, 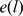 represents the normalized elution profile of the peptide at the 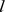
th scan, and 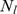 represents a noise vector added on 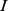 isotope positions. We can see that the observed intensities of a peptide, 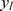, only changes in the scale 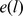, but not in the relative intensity pattern 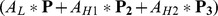 on different scans during the elution process.

If we consider all scans within the elution time of a peptide, then its feature can be represented by a two dimensional data matrix 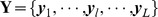, which has 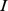 columns and 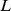 rows, representing the number of isotope positions and scans respectively. If we further sum 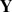 along the rows, we can get an overall intensity vector at all isotope positions:
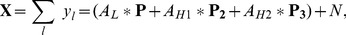
(4)where 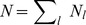.

A quantification algorithm generally takes 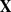 as the input, and estimate peptide abundances as 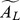, 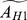, and 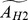. Based on these estimated abundances, we can construct an estimated peptide data model as 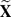 [25],
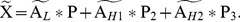
(5)


Elaborate models that consider the effect of 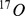 [20]–[22] can be employed for more accurate HLR calculation. However, since the primary focus of this paper is on BD and MFC, we have not expand our discussion in this direction.

Finally, the peptide HLR can be estimated as 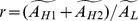.

If two observed features of a peptide are 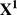 and 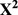, and based on which two HLR estimations are 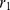 and 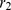, then the log-ratio-difference (LRD) is defined as 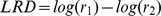.

## Approaches

Given a peptide with sequence, mass, and charge values from the input list, we start the quantification process by extracting its relevant XICs at different isotope positions as in (1). The goal of LC peak boundary detection is to find elution time intervals within which, the LC peaks on all XICs can be grouped to peptide features with matching mass and charge values to that of the peptide of interest. For this purpose, we first select an XIC at an isotope position with high abundance according to the predicted isotope pattern for both the unlabeled and the labeled peptide. Then we employ an initial LC peak picking algorithm on the selected XICs, which generates a list of candidate LC peak intervals. Then the consistency of intensity patterns within each LC peak interval will be checked to detect accurate boundaries. Subsequently, we perform a model fitness check step to filter out features that are incompatible with the mass and charge values of the peptide of interest. Features that passes these processing will be relayed to a quantification algorithm for HLR calculation. If elution time information is available, the feature with matching elution time will be reported. Otherwise, all features with matching mass and charge values will be reported.

**Figure 1:**
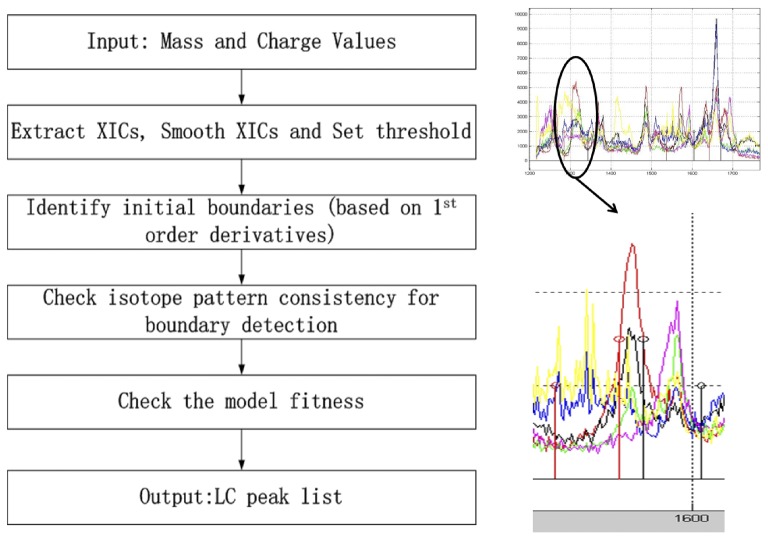
Flow Diagram of LC/MS Processing Steps. The right top panel shows a segment of XICs at different isotope positions of a peptide. There exists interference of co-eluting peptides. After processing, as shown in the bottom right panel, we determine the boundary of the LC peak (indicated by the stem pairs in the middle), that excludes segments which have been corrupted by signals of co-eluting peptides. The initial boundary (indicated by the lower pair of stems) includes a lot of interference from co-eluting peptides.

In Figure 1, we have shown the flow diagram of the proposed algorithm.

### Initial LC peak detection

The goal of initial LC peak detection is to find a list of candidate elution time intervals that could be matched to the peptide of interest. A procedure similar to that of MaxQuant [14] is employed.

1. Given a peptide's mass (m) at a charge state (z), determine its theoretical 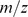 values at all 6 isotope positions, as in (1).
2. Predict the natural isotope distribution pattern 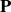, and select the highest isotope position, i.e. the index of 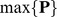, 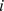. Usually 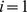 or 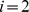. We consider the XICs at 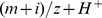 and 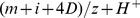 for the unlabeled and the labeled peptide respectively.
3. Employ a simple moving average (10 points) filter to smooth out the XICs selected. Since our initial LC peak detection does not require high accuracy, the requirement on the the smoothing algorithm is not high.
4. Combine the two selected XICs by summing them, and in this way, peaks generated by light and heavy peptides can be combined.
5. Estimate the background noise, and apply a threshold on the combined XIC. The threshold is set at three times the estimated background noise standard deviation.
6. For segments above the threshold, we derive first order derivatives and set initial LC peak boundaries at local minima. If an interval contains multiple LC peaks, the interval will be split to ensure that only one peak apex is contained in each interval.

### Boundary detection and model fitness check

The initial boundaries could include scans that have been corrupted by co-eluting peptides at certain isotope positions. We propose an additional peak boundary detection step to exclude interference.

1. Determine accurate LC peak boundaries of a peptide feature by checking the intensity pattern consistency. Since the observed intensities of a peptide, 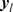 as defined in (3), only changes in the scale, but not in the relative intensity pattern during the elution process, we can detect interference by co-eluting peptides when the relative intensity pattern changes. The boundary detection process starts at the LC peak apex within the initial LC peak boundary. The basic assumption is that the intensity pattern at peak apex is not corrupted by co-eluting peptides. The normalized raw intensity values at all 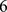 isotope positions in the scan of peak apex 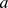, 
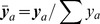
 will be used as a template. Then we move towards the beginning of the initial boundary one scan at a time, and compare the template pattern with that in the current scan 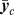. The difference between the intensity patterns is measured with KL distance [16] 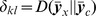, where given two normalized intensity vectors 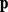 and 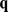, 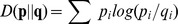. If 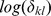 is smaller than 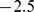, which indicates good matching, then the current scan will be included in the boundary. Otherwise, the current scan is deemed corrupted and will be excluded. The process stops once such a scan is encountered. We determine the threshold on 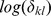 in the same way as that in MRCQuant [15]. Note that the KL distance is not used as a boundary detection criteria in MRCQuant as we proposed here, it is only used as a performance evaluation criteria, which is also used by MsInspect [10].
2. After boundary detection, all remaining elution intervals have consistent intensity patterns throughout the elution process, and can be considered as the candidate feature for the peptide of interest. Our next step aims at determining if these candidate features match in mass with the peptide of interest. To achieve this goal, we perform model fitness check by comparing the constructed data model, 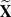 in (5), to the observed intensities 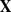. If there is a match, then the constructed data model, which is calculated based on the peptide's isotope pattern, should match to the observed data. Otherwise, the actual isotope pattern must have been generated by another peptide. Again KL distance between the normalized models is used as a measurement of deviation. A 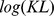 distance of less than −2.5 is considered as an indication of good match.

At the end of this process, a list of 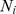 (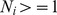) peptide features are identified for the 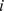
th mass and charge value.

In Figure 1, we show the flowgram of the proposed algorithm, and we plot one of the detected features. Six colors are used to plot six XICs at different isotope positions. We can see that the peak detection process successfully locates boundaries that exclude interference from co-eluting peptides.

### Quantification

After LC peak boundary detection and model fitness check, the identified peptide features can be further processed by quantification algorithms designed for 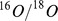 data. We employ a quantification algorithm to estimate the abundance of heavy and light labeled peptides, and calculate their ratios. We have surveyed the field and identified two popular methods: Yao's method [18] and the bilinear regression (BR) method [19].

Given 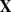 as defined in (4), Yao's method simply estimates the abundance of the light peptide using the intensity at the first isotope position. Subsequently, the expected intensity due to the unlabeled peptide is subtracted from the total intensity at the 3rd isotope position. The remaining value is used to estimate the abundance of the singly 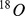 labeled peptide. After these two steps, the algorithm proceeds to estimate the total abundance of the doubly 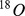 labeled peptide. This simple algorithm performs very well.

The BR method is a more sophisticated optimization method which aims at minimizing the mean square error between 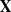 and 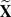. This implies that it considers the intensity at all isotope positions as independent. We employ both quantification algorithms after LC peak boundary detection and model fitness check to report final quantification results.

Employing more sophisticated quantification methods that consider the effect of 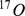 [20]–[22] could further improve the performance of 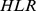 calculation.

### Accuracy evaluation by Normalized Mean Absolute Error (NMAE) in samples without predefined ratios

In experiment two, samples from two biological conditions are combined with equal amount of proteins. Without knowing the actual HLR, we can employ NMAE as an accuracy measure. The 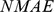 is defined as:
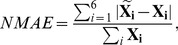
(6)where 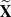 and 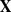 are the fitted and the observed intensities as defined before. Theoretically, if the abundance values 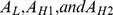 are estimated accurately by a quantification algorithm, then 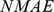 should be small. Thus 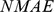 can be used as an accuracy measurement for quantification algorithms.

### Performance evaluation by Log Ratio Difference (LRD)

It is a general practice to evaluate the precision of a quantification algorithm by using samples with known ratios. However, the calculated variance of such samples comes from many sources including sample preparation, LC-MS instruments, and the algorithm itself. If we want to focus more on the variation caused by the algorithm, we can use LRD, in which, deviations caused by sample preparation are canceled out. Given the same accuracy in estimating the ratios (which is indicated by NMAE), and if the LRD is calculated between two replicates, then it reflects the sum variation caused by different runs of LC-MS, the instrument and the algorithm. When we split the scans within one LC peak into two parts, then the LRD reflects algorithm and instrument variation only. Ideally LRDs should have zero mean and small variance.

## Results and Discussion

### Performance evaluation based on samples with predefined ratios

**Figure 2:**
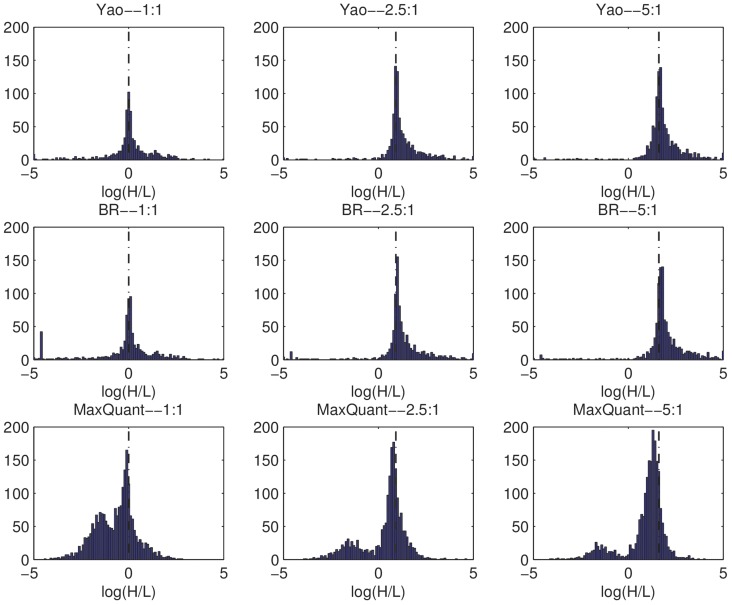
Histograms of measured 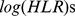 after applying BD and MFC. The vertical lines indicate the predefined ratios. We can see that MaxQuant returns biased ratio measurments while the proposed algorithm does not.

**Figure 3:**
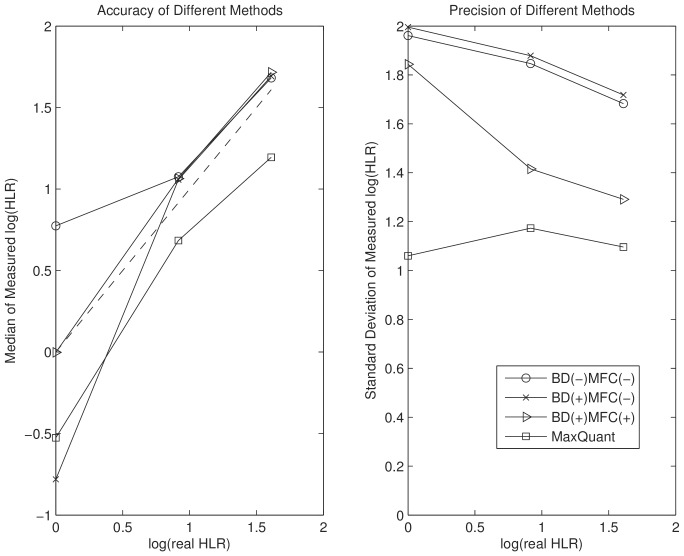
Comparison of accuracy and precision of different methods based median and standard deviation of 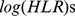.

In the first experiment, the HLRs are predefined as 1∶1, 2.5∶1, and 5∶1. We apply our proposed algorithm and compare it with MaxQuant. The histograms of the measured 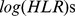 after BD and MFC are plotted in Figure 2, in which we can see that the histograms of MaxQuant 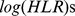 are far from Gaussian. We first try to compare performance using classical accuracy and precision measures, the median and the standard deviation of the 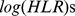, which are plotted in Figure 3. We consider three cases with or without the two proposed processing steps. If a step is applied, we indicate it by a plus sign. For example, (BD (+)) stands for the case that boundary detection is performed. The three cases are: 1. (BD (−) MFC (−)), 2. (BD (+) MFC (−)), and 3. (BD (+), MFC (+)). By comparing case one and case two, we can elucidate the effect of adding BD to the proposed algorithm. By comparing case two and case three, we can estimate the effect of MFC after boundary detection. These plots are generated based on Yao's method. The results based on the BR's method are similar. We can see that BD and MFC improve precision and accuracy significantly at all predefined ratios. However in Figure 3, we cannot tell if the proposed algorithm performs better than MaxQuant due to the phenomenon of bias-variance trade-off [26]. MaxQuant reports larger bias but smaller standard variation on 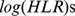.

**Figure 4:**
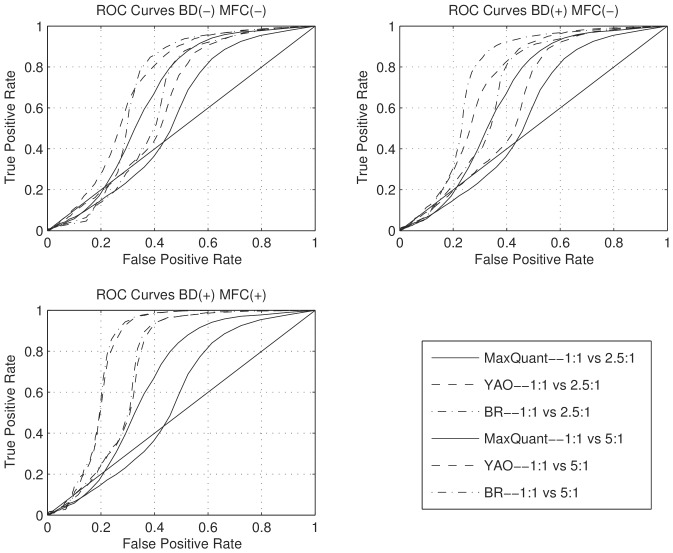
ROC Curves with (+) or without (−) boundary detection (BD) and model fitness check (MFC).

Since precision and accuracy cannot determine the separation of histograms (i.e. the resolution of measurement algorithms) when there exists bias-variance trade-off, we further employ receiver operating characteristic (ROC) curves to measure the separation between histograms. Specifically, we investigate the separation between the 1∶1 and the 2.5∶1/5∶1 histograms. At a given threshold 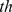, if a peptide has a 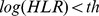, or 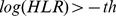, then the peptide ratio is considered to be 1∶1. The ROC curve plots the percentage of samples correctly identified as 2.5∶1/5∶1 (true positive rate) at different false positive rates (percentage of 1∶1 ratios identified as 2.5∶1/5∶1) as 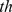 varies. If an algorithm separates histograms better, then at a given false positive rate, the true positive rate should be higher. This is a systematic way for comparing the resolution of different quantification methods. The results are summarized in Figure 4. We can see that combining BD and MFC improves the performance significantly.

BD and MFC do lead to reduced quantification coverage. The number of quantified peptides in samples with predefined ratios are compared to that of MaxQuant in Table 1. We can see that these filtering steps lead to improved ROC curves with a cost on quantification coverage.

**Table 1:** Number of quantified peptides on samples with predefined ratios with (+) or without (−) boundary detection (BD) and model fitness check (MFC).

	MaxQuant	BD (−) MFC (−)	BD (+) MFC (−)	BD (+) MFC (+)
1:1 Sample	2228	1923	1791	750
2.5:1 Sample	1827	1540	1441	903
5:1 Sample	2076	158	1456	1067

The histograms of measured log ratios in case one, (BD (−) MFC (−)), and case two, (BD (+) MFC (−)), are plotted as Figure S1 and Figure S2 in document S1.

### The validation of using NMAE and LRD standard deviation as performance measures based on samples with predefined ratios

**Figure 5:**
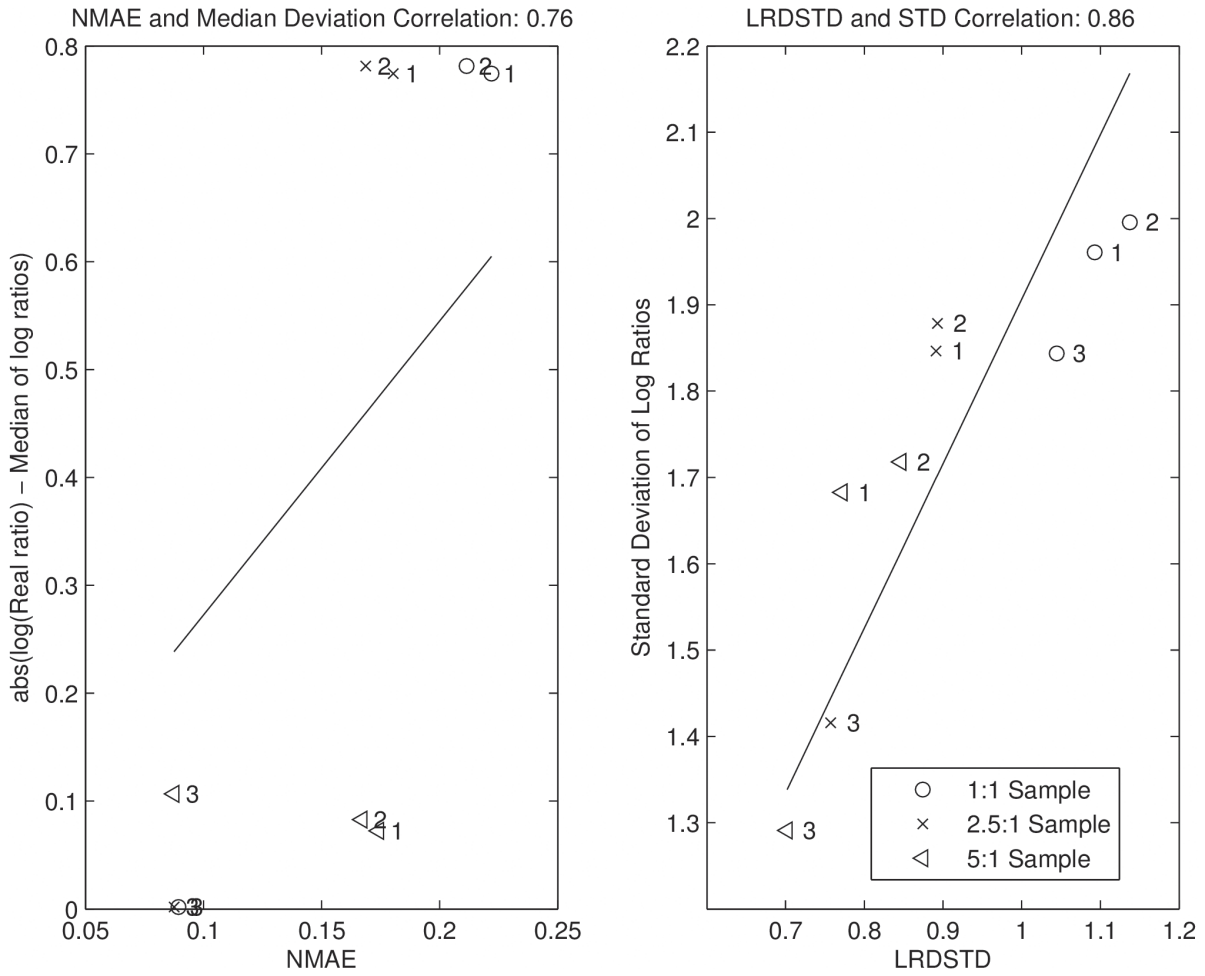
The correlation between the proposed precision and accuracy measures (NMAE and LRDSTD), and classical ones (median deviation and standard deviation of 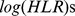). All three samples with predefined ratios are evaluated in all three cases: 1. BD (−) MFC (−); 2. BD (+) MFC (−); and 3. BD (+) MFC (+).

We intend to use NMAE and LRD to examine the performance of the proposed algorithm on samples without predefined ratios, where classical precision and accuracy measures cannot be calculated. But before we apply them, we first test if they are appropriate substitutes. We calculate NMAE and and the standard deviation of LRD (LRDSTD) based on samples with predefined ratios. To calculate LRD, we partition the scans within peptide features into two parts depending on if their scan numbers are even or odd. Subsequently, we quantify two parts separately to get their LRDs. In Figure 5, we plot NMAE and LRDSTD against the median and standard deviation of 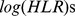 respectively. We can see that NMAE and LRDSTD regress to classical measures with correlations of 0.76 and 0.86. The establishment of NMAE and LRDSTD as substitute precision and accuracy measurements is very important since these two measures can be used in any experiments for performance evaluation.

In order to understand the effect of the proposed algorithm on LC peaks with different intensities, we partition considered features into three groups: the lower intensity group (bottom 20%), the middle intensity group (middle 60%), and the upper intensity group (top 20%). The analysis is carried out on these groups in addition to the three cases with or without BD and MFC using NMAE and LRDSTD.

**Figure 6:**
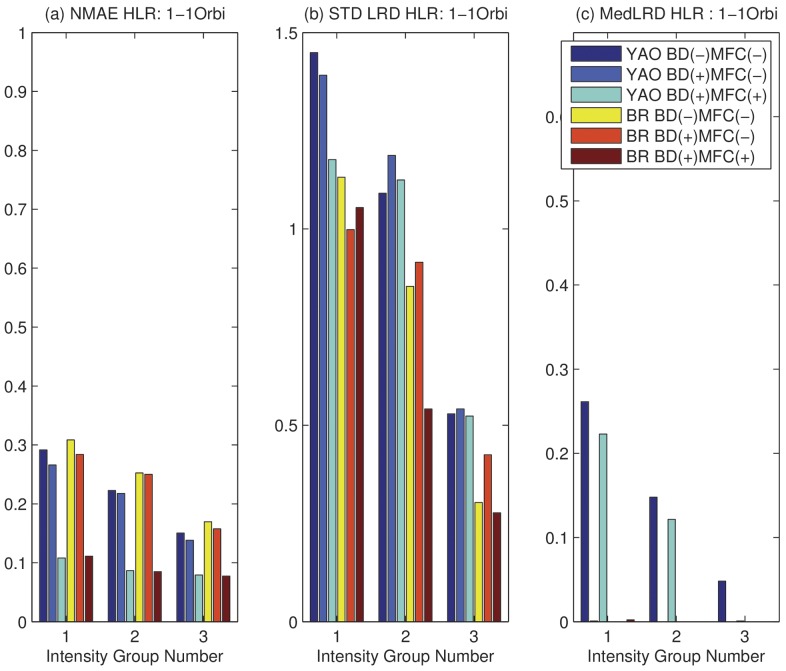
NMAE and LRD on Orbitrap 1∶1 sample with (+) or without (−) boundary detection (BD) and model fitness check (MFC) on three intensity groups. Intensity Group 1: lower 20%; 2: middle 60%; 3: upper 20%. (a) Normalized Mean Absolute Error (NMAE). (b) STD of LRD. (c) Median of LRD.

In Figure 6 (a), we compare 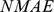 on the 1∶1 sample. We can see that, MFC and BD reduce 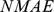 in all cases across all intensity groups. In Figure 6 (b), we compare the LRDSTD. We can see that there is a slight increase of LRDSTD after BD, but a significant reduction of LRDSTD after MFC. This is understandable because BD reduces the total number of scans used for quantification, which may lead to slight increase in LRDSTD. Given that BD reduces NMAE but increases LRDSTD, we can see that BD causes a bias-variance tradeoff. The overall effect is reflected by the improvement on ROC curves due to BD. MFC greatly reduces both NMAE and LRDSTD, because it removes peptides with wrongly assigned mass as well as peptides with significant interference. In Figure 6 (c), we can see that there is little difference in reported median of LRD except the case of using Yao's method on low and medium intensity groups. The median of LRD are expected to be around zero. Overall, on Orbitrap data with pre-defined ratios, there is a significant performance improvement in NMAE and LRDSTD.

NMAE and LRDSTD analysis on the 2.5:1 and 5:1 samples are shown in Figure S3 and Figure S4 in document S1, which largely agree with the results of the 1∶1 sample.

### Effect of accurate LC peak boundary detection and 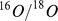 model fitness check on TOF and Orbitrap Datasets with two conditions

**Figure 7:**
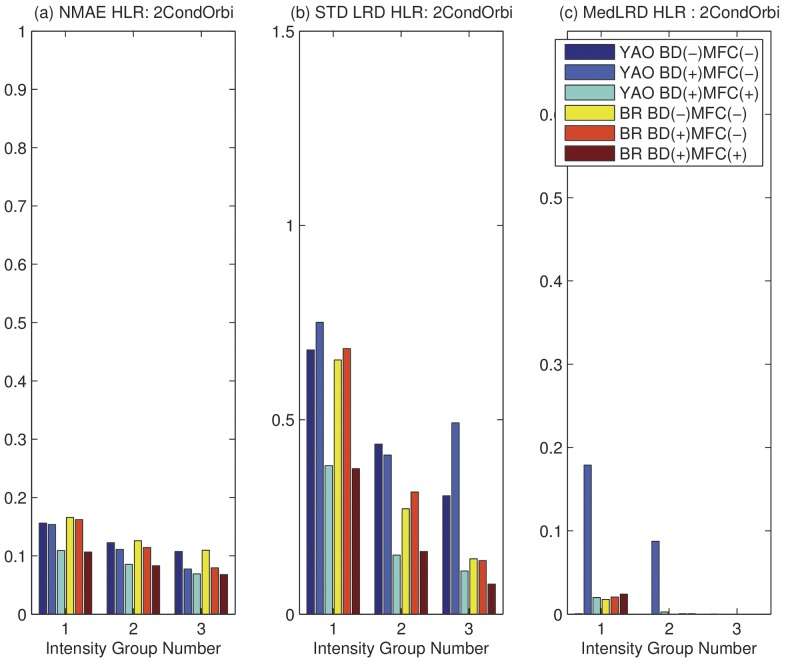
NMAE and LRD on sample with two conditions on Orbitrap with (+) or without (−) BD and MFC with three intensity groups. Intensity Group 1: lower 20%; 2: middle 60%; 3: upper 20%. (a) Normalized Mean Absolute Error (NMAE). (b) STD of LRD. (c) Median of LRD.

We have verified that NMAE and LRDSTD can reflect the improvement due to BD and MFC on samples with pre-defined ratios, now we want to see if similar improvement on these two measures can be obtained based samples without pre-defined ratios, which are relevant for real biomarker discovery projects. We first perform LRD and NMAE analysis on one replicate in experiment two collected on Orbitrap (OrbiR1) and TOF (TOFR1). The results on the Orbitrap data is shown in Figure 7. We can see that the results correlate well with those shown in Figure 6 for samples with pre-defined ratios. Significant performance improvement can be achieved by employing BD and MFC in common biological experiments. The LRD and NMAE analysis on the TOF data is shown as Figure S5 in document S1.

In document S1, we have also shown the NMAE and LRD analysis when considering two replicates collected on Orbitrap (OrbiR1/OrbiR2) in Figure S6. After processing each replicate separately, we obtain a union of 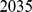 tandem MS identified peptides. Commonly identified peptides are considered as corresponding ones, based on which, LRDs can be calculated. Note that finding corresponding pairs based on tandem MS reduces the total number of considered peptides from 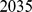 to 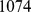. As a result, while strong performance improvement is shown for BD, no performance improvement is evident for MFC, whose filtering effect has been masked by the process of finding corresponding pairs using tandem MS. In practice, MFC cannot be replaced because a lot of peptides are not commonly identified in both replicates.

### Conclusion

In this paper, we propose to add two processing steps: LC peak boundary detection and model fitness check for 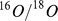 labeled LC-MS data processing. The performance of the algorithm is evaluated on samples with pre-defined ratios from cells in the same condition, and from cells in different biological conditions. We employ various measurements for evaluating the efficacy of the algorithm. On samples with pre-defined ratios, it is shown that the proposed algorithm improves the ROC curve performance significantly over that of MaxQuant. In experiment two, we further use NMAE and LRDSTD to evaluate the algorithm on samples from two different biological conditions. It is shown that significant reduction in NMAE and LRD (median and standard deviation of LRD), can be achieved due to LC peak boundary detection and model fitness check. The test is performed on data collected on both TOF and Orbitrap instruments.

The proposed algorithm is critical for reliable differential analysis for 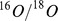 labeled data, which has a wide application in biomedical research.

### Supplementary information

For additional graphs, please see document S1. For MatLab Scripts and data processing output files, please see the following webpage:

http://compgenomics.utsa.edu/zgroup/boundarydetection/boundarydetection.html.

## Supporting Information

Document S1 **Document** **S1 contains supplementary figures (Figure S1 to Figure S6) used in the manuscript.**
(PDF)

Document S2 **Document** **S2 introduces how the experiment was performed.**
(PDF)
