# Supplementary material for: Accurate LC Peak Boundary Detection for 16 O/ 18 O Labeled LC-MS Data
Source: PLoS One. 2013 Oct 7;8(10):e72951. doi: 10.1371/journal.pone.0072951 (PMC3792097; doi:10.1371/journal.pone.0072951)
Supplement: Document S1 — contains supplementary figures (Figure S1 to Figure S6) used in the manuscript. (PDF) [file pone.0072951.s001.pdf]

Accurate LC Peak Boundary Detection for $^{16}O/^{18}O$ Labeled LC-MS Data Supplementary File

Jian Cui,[†] Konstantinos Petritis,[‡] Tony Tegeler,[‡] Brianne Petritis,[‡] Xuepo Ma,[†] Yufang Jin,[†] Shou-Jiang (SJ) Gao,[¶] and Jianqiu(Michelle) Zhang[*,§]

The University of Texas at San Antonio, San Antonio TX 78249 USA, Center for Proteomics, TGEN, Phoenix AZ 85004 USA, Keck School of Medicine, USC, Los Angeles, CA 90033 USA, and Electrical and Computer Engineering, UTSA, San Antonio, USA

E-mail: michelle.zhang@utsa.edu

References

(1) Cui, J.; Ma, X.; Chen, L.; Zhang, J. *BMC bioinformatics* **2011**, *12*, 439.

(2) others,, et al. *Proteomics* **2010**, *10*, 1150–1159.

[*] To whom correspondence should be addressed
[†] The University of Texas at San Antonio, San Antonio TX 78249 USA
[‡] Center for Proteomics, TGEN, Phoenix AZ 85004 USA
[¶] Keck School of Medicine, USC, Los Angeles, CA 90033 USA
[§] The University of Texas at San Antonio

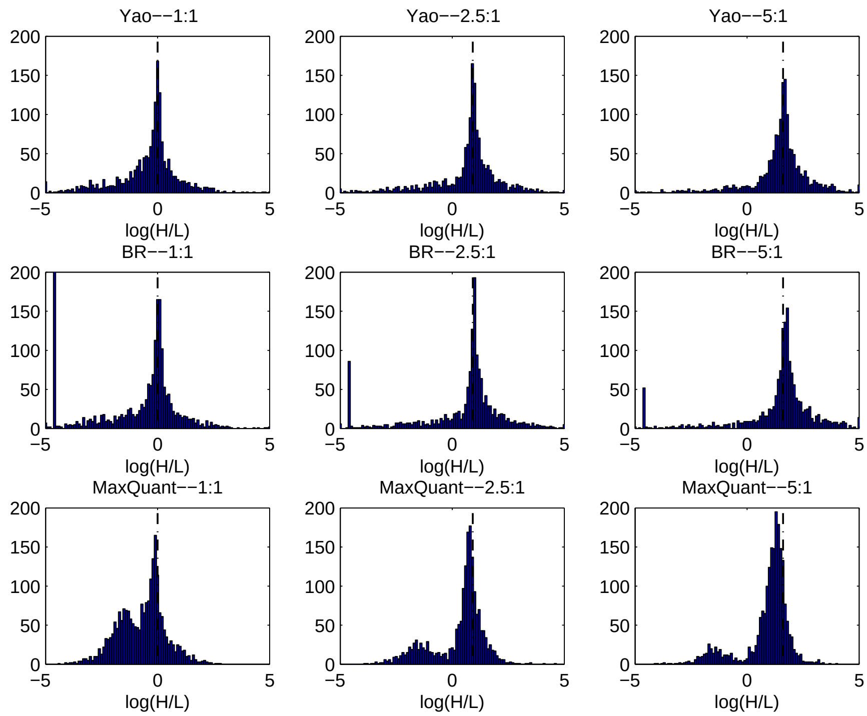

Figure S1: Histograms of measured log ratios without Boundary Detection and Model Fitness Check, BD(-) MFC(-). The vertical lines indicate the predefined ratios.

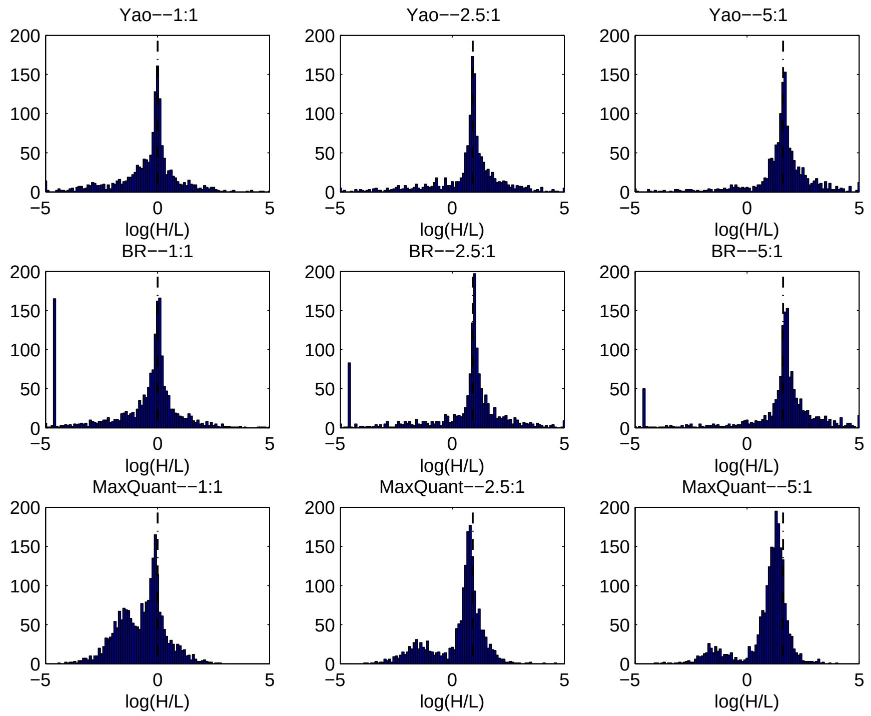

Figure S2: Histograms of measured log ratios after applying BD(+) but not MFC(-). The vertical lines indicate the predefined ratios.

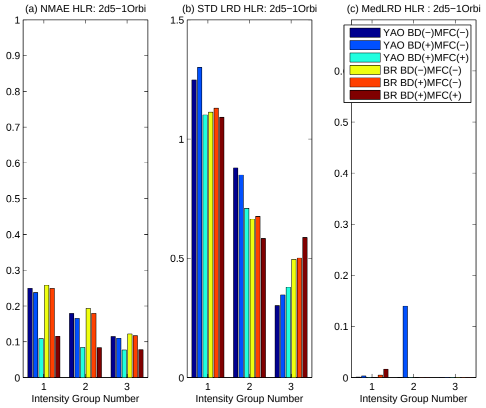

Figure S3: NMAE and LRD on Orbitrap 2.5:1 sample with(+) or without (-) boundary detection (BD) and model fitness check (MFC) on three intensity groups. Intensity Group 1: lower 20%; 2: middle 60%; 3: upper 20%. (a) Normalized Mean Absolute Error (NMAE). (b) STD of LRD. (c) Median of LRD

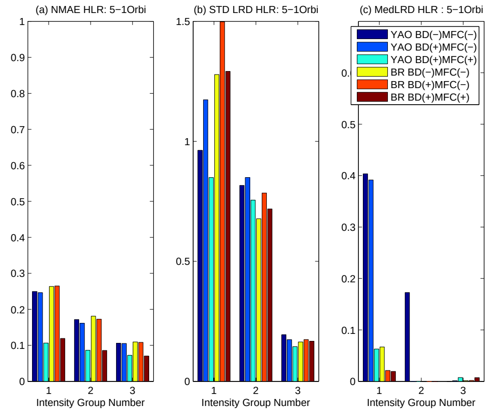

Figure S4: NMAE and LRD on Orbitrap 5:1 sample with(+) or without (-) boundary detection (BD) and model fitness check (MFC) on three intensity groups. Intensity Group 1: lower 20%; 2: middle 60%; 3: upper 20%. (a) Normalized Mean Absolute Error (NMAE). (b) STD of LRD. (c) Median of LRD

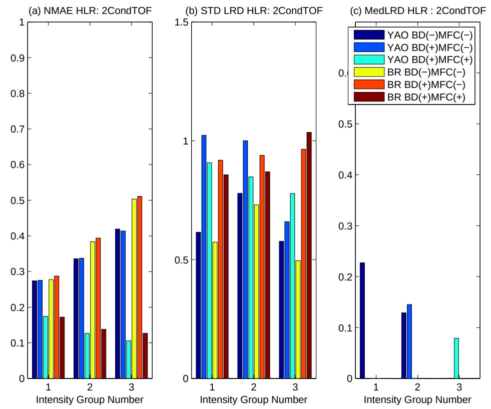

Figure S5: NMAE and LRD on TOF sample with two conditions with(+) or without (-) boundary detection (BD) and model fitness check (MFC) on three intensity groups. Intensity Group 1: lower 20%; 2: middle 60%; 3: upper 20%. (a) Normalized Mean Absolute Error (NMAE). (b) STD of LRD. (c) Median of LRD. We can see that MFC provids significant performance improvement in TOF.

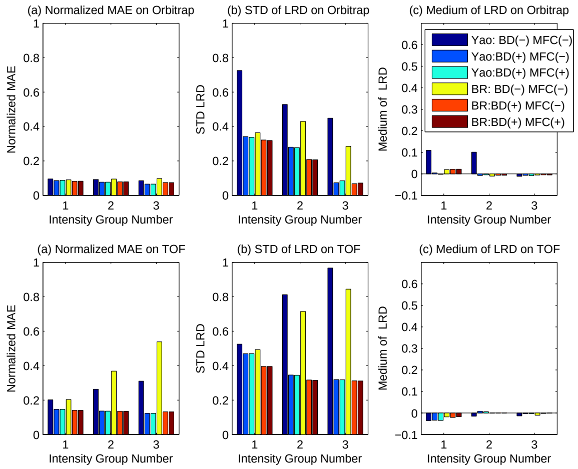

Figure S6: NMAE and LRD on samples with 2 conditions with(+) or without (-) boundary detection (BD) and model fitness check (MFC) on three intensity groups. Intensity Group 1: lower 20%; 2: middle 60%; 3: upper 20%. (a) Normalized Mean Absolute Error (NMAE) on Orbitrap. (b) STD of LRD on Orbitrap. (c) Median of LRD on Orbitrap (d)Normalized Mean Absolute Error (NMAE) on TOF.(e) STD of LRD on TOF. (f)Median of LRD on TOF. In this fiture we plot the NMAE and LRD analysis based on peptides that has corresponding features in another technical replicates. On the Orbitrap data, we first obtain a union of 2035 peptides identified through tandem MS. For the sake of evaluating the LRD between corresponding features in two replicates (OrbiR1,OrbiR2) , we first process each replicate separately, and identify corresponding features by looking for common peptides identified by tandem MS in both replicates. We find 1074 corresponding pairs. For the (TOFR1, TOFR2), we identify corresponding features by an algorithm similar to that reported in.[1] We can see that due to the requirement that these peptides must be corresponding features, the filtering effect of MFC is no longer obvious, and BD is the main factor in performance improvement. The analysis is performed based on peptides identified through the Trans-Proteomic Pipeline (TPP)[2] based on the Orbitrap dataset (OrbiR1, OrbiR2). International Protein Index (IPI) human database version 3.68 is selected as the source of protein sequences. For X!Tandem, the parent mass and fragment ions are searched with maximal mass errors of 7ppm and 0.5 dalton respectively. Methionine oxidation and n-terminal acetylation are considered as variable modifications, and cysteine carbamidomethylation is selected as the fixed modification. The modification mass of the C is set to 57.021464, and the potential modification mass of M is set to 15.994915. The input of the cleavage C-terminal mass change is set to 21.01. In database search, the minimum length of peptide is set to 6, and the maximum missed cleavage sites is set to 2. Finally Peptide-Prophet is used to validate the search results, and peptides with probability > 0.90 are exported into a text file for further quantification analysis.
