# Supplementary material for: Accurate LC Peak Boundary Detection for 16 O/ 18 O Labeled LC-MS Data
Source: PLoS One. 2013 Oct 7;8(10):e72951. doi: 10.1371/journal.pone.0072951 (PMC3792097; doi:10.1371/journal.pone.0072951)
Supplement: Document S2 — introduces how the experiment was performed. (PDF) [file pone.0072951.s002.pdf]

Liquid Chromatography Conditions

Strong Cation Exchange

UPLC system:	ACQUITY UPLC System
Column:	PolyLC Inc. PolySULFOETHYL A Column 100 x 2.1 mm, 3 μM, 300Å
Column temp:	35 °C
Flow Rate:	200 uL/min
Mobile phase A:	10 mM ammonium formate, 25% acetonitrile, pH 3.0
Mobile phase B:	500 mM ammonium formate, 25% acetonitrile, pH 3.0
Step-elution gradient:	Initial 0% B, 0 to 20% of mobile phase B in 40 min, 20 to 50% of mobile phase B in 10 min, 50 to 100% of mobile phase B in 5 min, and held for 2.5 min

1D Liquid Chromatography

UPLC system:	nanoACQUITY UPLC System
Column:	nanoAcquity UPLC BEH130 $C_{18,}$ 100 μM x 100 mm, 1.7 μM (p/n 186003546)
Column temp:	35 °C
Flow Rate:	500 nL/min
Mobile phase A:	0.1% FA in water (pH 2.4)
Mobile Phase B:	0.1% FA in acetonitrile
Gradient elution:	Initial 3.0% B, 3 to 7% of mobile phase B in 1 min, 7 to 25% of mobile phase B in 72 min, 25 to 45% of mobile phase B in 10 min, 45 to 95% of mobile phase B in 0.5 min, and held for 1 min

2D Liquid Chromatography

UPLC system:	nanoACQUITY UPLC System with 2D Technology and on-line dilution

First LC Dimension (Operating at pH 10.0)

Column:	Xbridge™ BEH300 $C_{18,}$ 5 um, 300 μM x 50 mm, NanoEase™ (p/n 186003682)
Flow Rate:	1.5 uL/min
Mobile phase A:	10 mM ammonium formate in water (pH 10)
Mobile phase B:	Acetonitrile
Step-elution gradient:	A 5-step elution gradient in the first dimension at pH 10 was undertaken. The percentage of mobile phase B in each step is: 10.8, 14.0, 16.7, 20.4, and 65.0% respectively
Diluting solution:	99.0% 0.1 FA in water and 1.0% 0.1 FA in Acetonitrile, 15 uL/min flow rate
Trap column:	Symetry $C_{18,}$ 180 μM x 20 mm, 5 um (p/n 186003514)

Second LC Dimension (Operating at pH 2.4)

Column:	nanoAcquity UPLC BEH130 $C_{18,}$ 100 μM x 100 mm, 1.7 μM (p/n 186003546)
Column temp:	35 °C
Flow Rate:	500 nL/min
Mobile phase A:	0.1% FA in water (pH 2.4)
Mobile Phase B:	0.1% FA in acetonitrile

Gradient elution:	Initial 3.0% B, 3 to 7% of mobile phase B in 1 min, 7 to 25% of mobile phase B in 72 min, 25 to 45% of mobile phase B in 10 min, 45 to 95% of mobile phase B in 0.5 min, and held for 1 min

Mass Spectrometry Conditions

**Mass Spectrometer System:**	ETD-LTQ-Orbitrap Velos
Spray source:	Thermo nanospray source coupled with a PicoTip fused silica spray tip (360mm OD, 20mm ID, 10mm diameter emitter orifice, New Objective, Woburn, MA, USA).
Acquisition Time:	95 min
m/z range:	400 - 4000 m/z
ESI spray voltage:	2.5 kV
Source temp:	300 °C
Resolution:	60,000
Auto gain control:	1,000,000
Number of ions for CID	Top fifteen
Dynamic exclusion repeat counts:	1 sec
Dynamic exclusion repeat duration:	30 sec
Dynamic exclusion duration:	60 sec

**Mass Spectrometer System:**	SYNAPT G2
Spray source:	Waters nanospray source coupled with a PicoTip fused silica spray tip (360mm OD, 20mm ID, 10mm diameter emitter orifice, New Objective, Woburn, MA, USA)
Acquisition Time:	95 min
m/z range:	50 - 2000 m/z
ESI spray voltage:	3.0 kV
Cone voltage:	40 V
Source temp:	120 °C
MS^E Low-energy:	4 eV (fixed)
MS^E High-energy:	Collision energy ramp between 15 and 35 eV

Data Collection

Experiment one sample preparation

In experiment one, samples from one Homo sapiens cell line (not specified by the providers) were divided into two groups. The two groups of samples were lysed in 8 M urea, and 50 mM ammonium bicarbonate (pH 8.3). The lysates were subjected to centrifugation at 13,000 rpm for 20 minutes and the supernatants were collected. The two samples were then denatured in 8 M urea, reduced using 10 mM dithiothreitol (DTT), alkylated with 30 mM iodoacetamide, and

digested with trypsin (using an enzyme to protein ratio of 1:50) at 37 °C overnight. The samples were desalted with Sep-Pak cartridges, separated into two tubes and dried in a speedvac. The first sample was resuspended in 100 mL 18O-water (Purity > 98%) containing 50 mM ammonium bicarbonate, 10 mM calcium chloride, and trypsin (1 to 50 w/w trypsin:peptide) pH 7.8. The second sample was treated in the same manner except that the 18O-water was replaced with purified 16O-water. After incubation with shaking at 450 rpm for 5 hours at 37 °C the labeling reaction was terminated by first boiling the sample for 10 minutes and then adding 5 mL of formic acid to further inhibit any residual trypsin activity. A bicinchoninic acid (BCA) assay was performed to determine peptide concentration. The two samples were combined equally or in selected ratios (1:1, 2.5:1, and 5:1 Heavy/Light) and were subjected to reverse phase liquid chromatography (LC) followed by ETD-LTQ-Orbitrap Velos mass spectrometry (MS) analysis (see Experimental section for 1D LC and ETD-LTQ-Orbitrap Velos MS conditions).

Experiment two sample preparation

In experiment two, the samples from Human embryonic kidney 293T cells were cultured in Dulbecco's modified Eagle's medium with 10% fetal bovine serum (FBS). One group was transfected with an expression vector expressing microRNA-K1 of Kaposi's sarcoma-associated herpesvirus (KSHV) while the control group was transfected with a vector for 48 h.[12] The sample preparation on these two groups of samples is the same as in experiment one. Equal amounts of 16O and 18O labeled samples were combined to obtain one sample. Two hundred micro grams of combined sample was fractionated into four fractions using strong cation exchange (SCX) (see Experimental section strong cation exchange for LC conditions). The four samples were then subjected to reverse phase-reverse phase LC followed by ETD-LTQ-Orbitrap Velos MS and SYNAPT G2 MS analysis (see Experimental section for 2D LC and MS conditions).
